# Calcitriol supplementation accelerates the recovery of patients with tuberculosis who have vitamin D deficiency: a randomized, single-blind, controlled clinical trial

**DOI:** 10.1186/s12879-022-07427-x

**Published:** 2022-05-05

**Authors:** Youli Wen, Lian Li, Zhiping Deng

**Affiliations:** 1 Department of Respiratory Medicine, Zigong First People’s Hospital, Zigong, 643000 Sichuan Province People’s Republic of China; 2 Department of Internal Medicine, Department of Obstetrics, Shenzhen Baoan Women’s and Children’s Hospital, Jinan University, No. 56 Yulv Road, Baoan District, Shenzhen, 518102 Guangdong Province People’s Republic of China

**Keywords:** Vitamin D, 25-(OH) D, Tuberculosis, CD4^+^ T cells

## Abstract

**Background:**

This study aimed to evaluate whether calcitriol supplementation enhances CD4^+^ T cell count and prognosis in patients with tuberculosis and low 25(OH)D levels.

**Methods:**

This randomized controlled clinical trial enrolled treatment-naïve patients with tuberculosis admitted to Zigong First People’s Hospital (June 2016 to April 2017). The patients were grouped as the serum 25(OH)D ≥ 75 nmol/L (Normal-25(OH)D) and 25(OH)D < 75 nmol/L (Low-25(OH)D) groups. The Low-Vit-D group was randomized to the Low-25(OH)D and Low-25(OH)D-Calcitriol groups. All patients were treated with the 2HRZE/4HR regimen. The patients in the Low-25(OH)D-Calcitriol group received calcitriol 0.25 µg bid. The main endpoints were CD4^+^ T cell count during treatment, time to sputum culture conversion, time to 50% lesion absorption, and a 6-month cure rate.

**Results:**

This study included 30 patients in each group. Baseline 25-(OH) D levels and CD4^+^ T cell counts were higher in the Normal-25(OH)D group than in the Low-25(OH)D and Low-25(OH)D-Calcitriol groups (25(OH)D: 79.3 ± 3.4 vs. 37.8 ± 13.4 vs. 11.9 nmol/L, *P* < 0.05; 671 ± 287 vs. 200 ± 110 vs. 194 ± 119 cell/µL, *P* < 0.05). 25-(OH) D levels and CD4^+^ T cell counts increased in the Low-25(OH)D-Calcitriol group during treatment to reach higher levels than in the Low-25(OH)D group at 1, 4, 8, and 24 weeks (all *P* < 0.05). Compared with the Low-25(OH)D group, the Low-25(OH)D-Calcitriol group showed shorter time to sputum culture conversion (3.2 ± 1.4 vs. 5.9 ± 2.5 days, *P* < 0.001) and time to 50% lesion absorption (7.4 ± 1.5 vs. 10.9 ± 4.0 days, *P* = 0.014) and similar to those in the Normal-25(OH)D group (3.1 ± 1.2 and 7.3 ± 1.5 days, respectively. The cure rate was 86.7% in the Low-25(OH)D group and 96.7% in the two other groups.

**Conclusions:**

Calcitriol supplementation can elevate CD4^+^ T cell levels, shorten the time to sputum culture conversion, and accelerate lesion absorption in patients with tuberculosis and 25(OH)D deficiency.

*Trial registration* The study is registered at the Chinese Clinical Trial Registry (ChiCTR2000039832)

## Background

Tuberculosis (TB) is a global health problem and the leading cause of death due to infectious diseases [1]. There were around 10 million cases of incident TB and 1.5 million TB-related deaths in 2018 [2], which illustrates the high burden of this disease worldwide. TB is a bacterial infection caused by *Mycobacterium tuberculosis*, which is spread from person to person via microscopic droplets released into the air [3]. TB can involve any organ but affects the lungs in 75% of cases [4]. Pulmonary TB generally presents with fever, cough, hemoptysis, and weight loss [5], but some patients have nonspecific symptoms. Investigations used for the diagnosis of TB include chest X-ray, computed tomography (CT), and sputum microscopy, culture, and nucleic acid amplification test [6]. TB is treated with a combination of antimicrobial agents such as isoniazid, rifampicin, ethambutol, and pyrazinamide [4].

25(OH)D deficiency is a common health problem in many countries [7, 8]. 25(OH)D plays a critical role in bone metabolism and growth but might also have immunomodulatory actions [9]. Interestingly, there is evidence that 25(OH)D deficiency might be associated with an increased risk of bacterial infections such as community-acquired pneumonia [10] and respiratory tract infections in children [11]. Notably, a recent prospective study reported that 25(OH)D deficiency was associated with a higher risk of incident TB in HIV-positive people [12]. Furthermore, studies indicated that patients with TB have lower levels of 25(OH)D than controls [13–15]. Calcitriol supplementation was found to increase the proportion of sputum smear/culture conversions in patients with TB [16] and shorten the time to sputum culture conversion in patients with multidrug-resistant pulmonary TB [17] or single nucleotide polymorphisms (SNPs) in 25(OH)D pathway genes [18, 19]. On the other hand, some studies did not observe any effects of calcitriol supplements on the risk of TB infection [20, 21]. Therefore, additional research is needed to establish whether calcitriol supplementation has a role as adjuvant therapy in patients with TB.

CD4^+^ T helper 1 (Th1) cells play a critical role in the immune response against intracellular pathogens such as *Mycobacterium tuberculosis* [22]. Induced Th1 cells synthesize interferon-γ, which activates macrophages to destroy intracellular pathogens [23]. A low CD4^+^ T lymphocyte count is associated with more severe disease and a poorer prognosis in patients with TB [24, 25]. There is increasing evidence that 25(OH)D influences the function of CD4^+^ T cells [26, 27]. Epidemiologic studies have shown that the incidence of active TB is substantially increased in patients who are infected with human immunodeficiency virus (HIV) and have decreased CD4^+^ T cell levels [28, 29]. Moreover, 25(OH)D deficiency might accelerate the decline in CD4^+^ T cell count in people living with HIV [30]. In addition, calcitriol supplementation was shown to improve CD4^+^ T cell recovery in HIV-positive adults undergoing antiretroviral therapy [31]. Still, there are limited published data regarding the effects of calcitriol supplementation on CD4^+^ T lymphocyte levels in patients with TB.

We hypothesized that calcitriol supplementation would enhance CD4^+^ T cell levels in patients with TB and improve their response to therapy. Therefore, the aim of this study was to measure 25(OH)D levels in patients with TB and evaluate the impact of calcitriol supplementation on the immune function and prognosis of patients with TB and low 25(OH)D levels.

## Methods

### Study design and participants

This randomized controlled clinical trial included patients with TB admitted to the Respiratory Medicine, Tuberculosis and Infectious Diseases Department of Zigong First People’s Hospital, Sichuan, China, between June 2016 and April 2017. The inclusion criteria were (1) 18–70 of age, (2) etiologically-confirmed TB in accordance with the WS 288-2008 Diagnostic Criteria for Pulmonary Tuberculosis and (after data collection) the WS 288-2017 Diagnostic Criteria for Pulmonary Tuberculosis published by the Ministry of Health, People’s Republic of China, and (3) treatment-naïve. The exclusion criteria were (1) clinically suspected cases of TB, (2) airway involvement of TB as defined by the WS 288-2008 Diagnostic Criteria for Pulmonary Tuberculosis, (3) non-compliance with therapy, (4) failure to receive regular follow-up, (5) allergy to anti-TB drugs, and (6) severe liver or renal dysfunction. This study was reviewed and approved by the Ethics Committee of Zigong First People’s Hospital (No.24, 2016). All participants were fully informed of the risks and provided written informed consent before enrollment. The study is registered at the Chinese Clinical Trial Registry (ChiCTR2000039832; registered on 11-11-2020).

### Calculation of sample size

The sample size was calculated using the equation $n=\frac{2\stackrel{-}{p}\stackrel{-}{q}{\left({Z}_{\alpha }+{Z}_{\beta }\right)}^{2}}{{\left({p}_{1}-{p}_{2}\right)}^{2}}$ [32]. Assuming a cure rate (the primary endpoint) of 99% in the intervention group, a cure rate of 75% in the control group, an α of 0.05, a 1:1 allocation between the Low-25(OH)D and Low-25(OH)D-Calcitriol groups, and a power of 0.8, the sample size was calculated to be 30/group.

### Groups and blinding

The participants were allocated to the serum 25(OH)D level ≥ 75 nmol/L (Normal-25(OH)D group) and < 75 nmol/L groups according to their level of serum 25(OH)D. Then, the participants with serum 25(OH)D levels < 75 nmol/L were randomized into the Low-25(OH)D and Low-25(OH)D-Calcitriol groups. Randomization was performed using a random number table and random number remainder grouping method. The allocation sequence was prepared into opaque sealed envelopes, and one envelope was opened sequentially upon enrollment and grouping into the Low-25(OH)D group. The investigators who collected and analyzed the data were blinded to the interventions.

### Intervention

The participants in the Normal-25(OH)D and Low-25(OH)D groups were treated with isoniazid, rifampin, pyrazinamide, and ethambutol for 2 months, followed by isoniazid plus rifampin for 4 months (2HRZE/4HR regimen) if they were hepatitis B-negative and had a normal liver function. The participants with hepatitis B or abnormal liver function received isoniazid, rifapentine, levofloxacin, and ethambutol for 2 months, followed by isoniazid plus rifapentine for 4 months (2HR_t_LE/4HRt). The participants in the Low-25(OH)D-Calcitriol group were given anti-TB medications as above and calcitriol soft capsules 0.25 µg bid (CP Pharmaceutical Group, Qingdao, China). The 25(OH)D levels were monitored at the start of therapy and at 1 week, 4 weeks, 2 months, and 6 months after starting the therapy, and the calcitriol supplementation was discontinued if the 25-(OH) D level reached the normal range (≥ 75 nmol/L). The patients were not instructed to use UV light or to change their outdoor activity habits.

### Measurement of CD4^+^ T cell count and 25-(OH) D levels

Peripheral venous blood was collected in an anticoagulant tube, and 100 µL of blood was incubated with 20 µL of antibody (CD45/CD4/CD8/CD3) in the dark at 18–26 °C for 20 min. Subsequently, 500 µL of OptiLyse C lysing solution was added, and the mixture was incubated in the dark at 18–26 °C for 20 min. Then, 3.0 mL of phosphate buffer was added, and the sample was centrifuged at 1500 rpm for 5 min. The supernatant was removed, and 500 µL of phosphate buffer was added to resuspend the cells. The cell concentration was adjusted to 10^6^–10^7^ cells/mL, and the frequency of CD4^+^ T cells was determined using a LIAISON-XL automatic chemiluminescence immunoassay analyzer (Beckman Coulter, Brea, CA, USA). The entire process was performed by laboratory physicians with 8 years of experience. The measurement of 25-(OH) D levels was carried out using a 25-(OH)D assay kit in accordance with the manufacturer’s instructions (DiaSorin, Saluggia, Italy).

### Tuberculosis score and sputum culture

The TB severity score was calculated according to the number of lung lobes involved (1 point for each lobe involved) and the presence or absence of cavities on chest CT (1 point for each cavity detected) [33]. A 50% lesion absorption was defined as a 50% reduction in the TB severity score or a 50% reduction in the total lesion area on chest CT. The sputum culture was performed using standardized clinical methods by the central hospital laboratory. The acid Lowenstein–Jensen medium was used.

### Endpoints and follow-up

All participants were followed up via telephone and outpatient clinic. The changes in the various indicators were assessed at 1 week, 4 weeks, 8 weeks, and 24 weeks after the start of anti-TB therapy and at the end of the study period. The primary endpoint was the cure rate after 24 weeks of anti-TB therapy. The criteria used to define cures were sputum culture conversion (indicating elimination of *Mycobacterium tuberculosis*) and lesion improvement on imaging (either complete absorption of the lesions with the cavities occluded for more than 24 weeks; or if residual cavities remained, sputum culture conversion was maintained for at least 1 year after the completion of treatment). The secondary endpoint was the change in the frequency of CD4^+^ T cells.

### Data collection

The baseline clinical data and evaluation indicators were extracted from the electronic medical records. The baseline clinical data included age, sex, body weight, smoking status, and alcohol consumption. The evaluation indicators included CD4^+^ T cell count, serum calcium, sputum culture result (positive or negative), chest CT findings (presence/absence of cavities), white blood cell count (WBC), platelet count (PLT), hemoglobin level, and serum levels of alanine transaminase (ALT), aspartate transaminase (AST), bilirubin, creatinine (CREA), and electrolytes. The evaluation indicators were assessed at baseline and at 1 week, 4 weeks, 8 weeks, and 24 weeks after the initiation of anti-TB therapy and at the end of the study. The culture was performed using standardized clinical methods by the central hospital laboratory. The acid Lowenstein–Jensen medium was used.

### Statistical analysis

The analyses were performed using SPSS 17.0 (IBM, Armonk, NY, USA). Continuous variables were presented as the mean ± standard deviation (SD). Normally distributed data were compared between groups using the t-test for independent samples (two groups) or one-way analysis of variance (ANOVA) with Dunnett’s post hoc test (three or more groups). Non-normally distributed data were compared between groups using the Mann–Whitney U-test (two groups) or Kruskal–Wallis test (three or more groups). Categorical data are presented as n (%) and were compared between groups using the Chi-squared test or the corrected Chi-squared test using a four-fold table. A two-tailed *P*-value < 0.05 was considered statistically significant.

## Results

### Baseline characteristics of the study participants

**Fig. 1:**
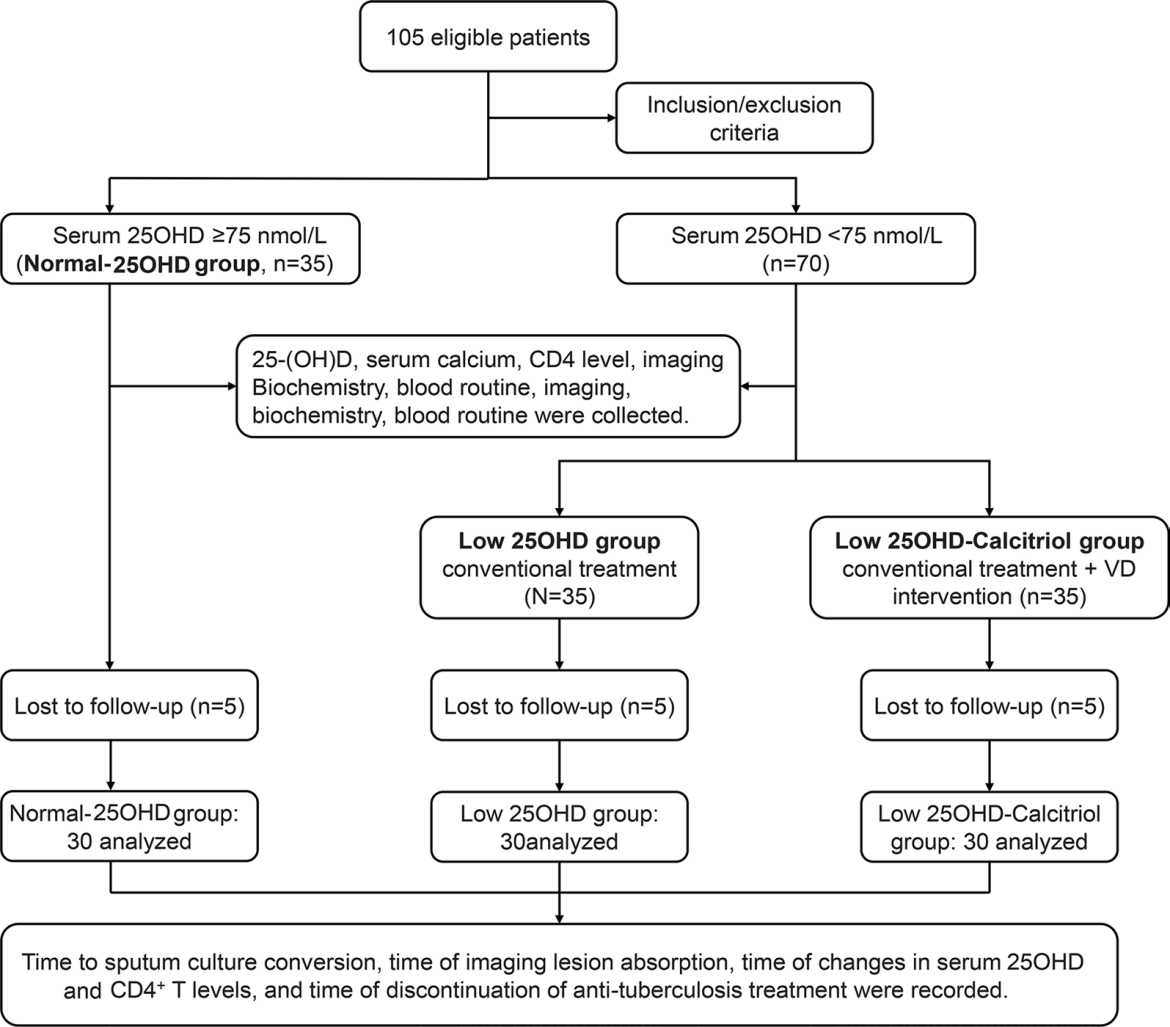
Patient enrollment in the study

Initially, 105 participants were enrolled, with 35 cases in each group. Five participants in each group did not complete the outpatient follow-up. Therefore, the final analysis included 30 participants in each of the three groups (Fig. 1). The baseline clinical characteristics of the study participants are shown in Table 1. Participants in the Normal-25(OH)D group were significantly younger than those in the Low-25(OH)D and Low-25(OH)D-Calcitriol groups (43.3 ± 14.0 years vs. 56.1 ± 14.0 years and 56.7 ± 14 years, respectively; *P* < 0.05), without significant difference between the Low-25(OH)D and Low-25(OH)D-Calcitriol groups (*P* > 0.05). The baseline serum 25-(OH) D levels of the participants were significantly higher (*P* < 0.05) in the Normal-25(OH)D group (79.3 ± 3.4 nmol/L) than that in the Low-25(OH)D (37.8 ± 13.4 nmol/L) and Low-25(OH)D-Calcitriol (39.3 ± 11.9 nmol/L) groups, without significant difference between the Low-25(OH)D and Low-25(OH)D-Calcitriol groups (*P* > 0.05). Notably, baseline CD4^+^ T cell count of patients was also significantly higher (*P* < 0.05) in the Normal-25(OH)D group (671.1 ± 286.9 cells/µL) than that in the Low-25(OH)D (199.7 ± 110.2 cells/µL) or Low-25(OH)D-Calcitriol group (193.9 ± 119.2 cells/µL), with no significant difference between the Low-25(OH)D and Low-25(OH)D-Calcitriol groups (*P* > 0.05). There were no statistically significant differences among the groups for the other characteristics (all *P* > 0.05). There were no differences among the three groups in the seasons of intervention.

**Table 1:** Baseline characteristics of the study participants

Characteristic	Normal-25(OH)D group (n = 35)	25(OH)D < 75 nmol/L		*P*
		Low-25(OH)D group (n = 35)	Low-25(OH)D-Calcitriol group (n = 35)	
Age (years)	43.3 ± 14.0	56.1 ± 14.0*	56.7 ± 14.0*	< 0.05
Sex				0.986
Male	20 (57.1%)	21 (60.0%)	22 (62.8%)	
Female	15 (42.9%)	14 (40.0%)	13 (37.2%)	
Body mass index (BMI) (kg/m^2^)	21.0 ± 1.4	19.8 ± 1.2	19.7 ± 1.2	0.955
Smoker	25 (71.4%)	26 (74.3%)	26 (74.3%)	0.988
Alcohol consumption	26 (74.3%)	27 (77.1%)	26 (74.3%)	0.987
Sputum smear at enrollment (n = 35/group)				
Positive	35 (100%)	35 (100%)	35 (100%)	
Negative	–	–	–	
Tuberculosis score	3.37 ± 1.14	3.42 ± 1.15	3.41 ± 1.15	0.971
Serum 25(OH)D level (nmol/L)	79.3 ± 3.4	37.8 ± 13.4*	39.3 ± 11.9*	< 0.05
White blood cell count (×10^9^/L)	6.2 ± 1.4	5.5 ± 1.1	5.4 ± 1.0	0.933
Hemoglobin (g/L)	109 ± 12	104 ± 10	105 ± 11	0.901
CD4^+^ T cell count (cells/µL)	671.1 ± 286.9	199.7 ± 110.2*	193.9 ± 119.2*	< 0.05
Chest radiography				0.289
Cavities present	16 (45.7%)	17 (42.9%)	16 (45.7%)	
Cavities absent	19 (54.3%)	18 (57.1%)	19 (54.3%)	
Drug sensitivity profile				
Multidrug-resistant	35 (100%)	35 (100%)	35 (100%)	
Non-multidrug-resistant	0 (0%)	0 (0%)	0 (0%)	
Treatment regimen				0.964
2HRZE/4HR	25 (71.4%)	24 (68.6%)	23 (65.7%)	
2HR_t_LE/4HR_t_	10 (28.6%)	11 (31.4%)	12 (34.3%)	

Data are presented as mean ± standard deviation or n (%)

*P < 0.05 vs. Normal-25(OH)D group

### Clinical outcomes

The cure rate (cavities absent) was not significantly different among the Normal-25(OH)D (29/30, 96.7%), Low-25(OH)D-Calcitriol (29/30, 96.7%), and Low-25(OH)D groups (26/30, 86.7%) (*P* = 0.289) (Table 2). The time to sputum culture conversion and time to 50% lesion absorption were significantly longer in the Low-25(OH)D group (5.9 ± 2.5 and 10.9 ± 4.0 days, respectively) than in the Normal-25(OH)D group (3.1 ± 1.2 and 7.3 ± 1.5 days, respectively, *P* < 0.05) (Table 2), and the time to sputum culture conversion and time to 50% lesion absorption of patients in the Low-25(OH)D-Calcitriol group were significantly shorter than in the Low-25(OH)D group (3.2 ± 1.4 vs. 5.9 ± 2.5 and 7.4 ± 1.5 vs. 10.9 ± 4.0 days, all *P* < 0.05) (Table 2), while were no significant differences compared with the Normal-25(OH)D group (*P* > 0.05).

**Table 2:** Outcomes after 6 months of treatment

Characteristic	Normal-25(OH)D group (n = 30)	25(OH)D < 75 nmol/L		*P*
		Low-25(OH)D group (n = 30)	Low-25(OH)D-Calcitriol group (n = 30)	
Time to sputum culture conversion (days)	3.10 ± 1.16	5.87 ± 2.46*	3.20 ± 1.35^#^	< 0.001
Time to 50% lesion absorption (days)	7.31 ± 1.49	10.85 ± 3.97*	7.38 ± 1.47^#^	0.014
Sputum smear				
Positive	0 (0%)	0 (0%)	0 (0%)	
Negative	30 (100%)	30 (100%)	30 (100%)	
Baseline chest radiograph				0.289
Cavities present	1 (3.3%)	4 (13.3%)	1 (3.3%)	
Cavities absent	29 (96.7%)	26 (86.7%)	29 (96.7%)	

Data are presented as mean ± standard deviation or n (%)

*P < 0.05 vs. Normal-25(OH)D group

^#^*P* < 0.05 vs. Low-25(OH)D group

### ^+^ T cell counts during therapy

**Fig. 2:**
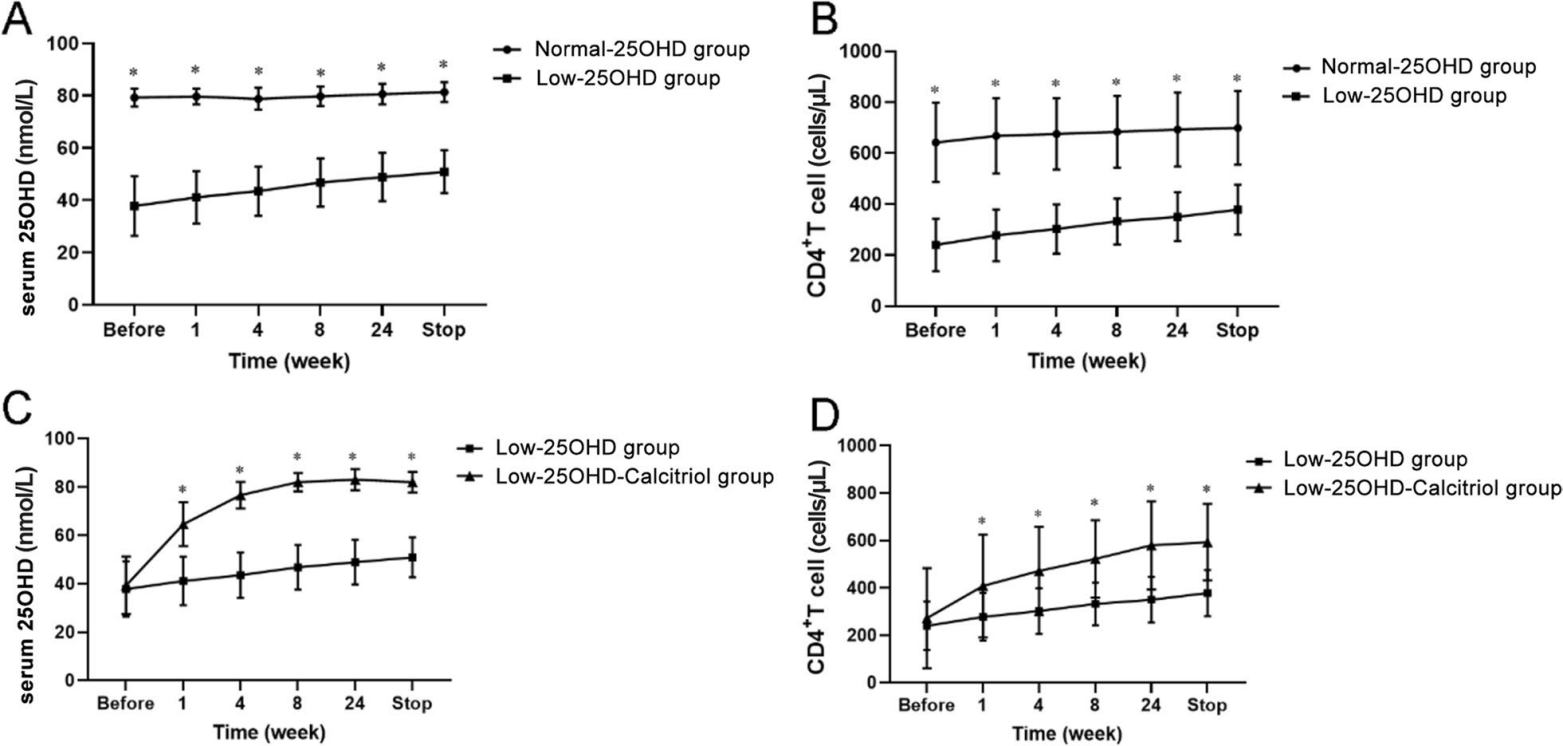
Changes in serum vitamin D and CD4^+^ T cell count at different time points. **A** Changes in serum vitamin D levels in the Normal-25(OH)D group and Low-25(OH)D group. **B** Changes in serum vitamin D levels in the Low-25(OH)D group and Low-25(OH)D-Calcitriol group. **C** Changes in CD4^+^ T cell levels in the Normal-25(OH)D group and Low-25(OH)D group. **D** Changes in CD4^+^ T cell levels in the Low-25(OH)D group and Low-25(OH)D-Calcitriol group. *P < 0.05

The 25-(OH) D levels and CD4^+^ T cell counts of the participants in the Normal-25(OH)D group remained relatively stable throughout the study period. Although the 25-(OH) D levels and CD4^+^ T cell counts in the Low-25(OH)D group exhibited increasing trends during conventional anti-TB therapy, their levels remained significantly lower than in the Normal-25(OH)D group at all time points (all *P* < 0.05) (Fig. 2 A, B).

The baseline 25-(OH) D levels were comparable between the Low-25(OH)D-Calcitriol and Low-25(OH)D groups, but the 25-(OH) D levels in the Low-25(OH)D-Calcitriol group increased substantially during treatment (which included calcitriol supplementation) and were significantly higher than in the Low-25(OH)D group at 1 week, 4 weeks, 8 weeks, 24 weeks, and the end of study (*P* < 0.05) (Fig. 2C). Notably, the CD4^+^ T cell count in the Low-25(OH)D-Calcitriol group also increased during the treatment period to levels that were significantly higher than in the Low-25(OH)D group at 1 week, 4 weeks, 8 weeks, 24 weeks, and the end of study (*P* < 0.05; Fig. 2D).

### Adverse events

The most common adverse events encountered in all three groups were elevated serum uric acid levels, gastrointestinal reactions, liver function abnormalities, reduced WBC count, and reduced PLT count. These adverse events of the participants in the three groups are listed in Table 3. Furthermore, all adverse events were mild, were relieved by symptomatic treatment, and did not affect the course of anti-TB therapy.

**Table 3:** Adverse events

Adverse events	Normal-25(OH)D group (n = 30)	25(OH)D < 75 nmol/L	
		Low-25(OH)D group (n = 30)	Low-25(OH)D-Calcitriol group (n = 30)
Abnormal liver function	5 (16.7%)	6 (20.0%)	5 (16.7%)
Elevated uric acid	19 (63.3%)	18 (60.0%)	20 (66.7%)
Gastrointestinal reactions (nausea and reflux)	10 (33.3%)	10 (33.3%)	11 (33.3%)
Decrease in white blood cell count	2 (6.7%)	2 (6.7%)	2 (6.7%)
Decrease in platelet count	1 (3.3%)	1 (3.3%)	1 (3.3%)

Data are presented as n (%)

## Discussion

A notable finding of the present study was that the baseline CD4^+^ T cell counts were significantly higher in the Normal-25(OH)D group than in the Low-25(OH)D and Low-25(OH)D-Calcitriol groups. Furthermore, the 25-(OH)D levels and CD4^+^ T cell counts increased in the Low-25(OH)D-Calcitriol group during treatment to reach levels that were significantly higher than in the Low-25(OH)D group at 1, 4, 8, and 24 weeks and the end of the study. Importantly, the time to sputum culture conversion and time to 50% lesion absorption in the Low-25(OH)D-Calcitriol group were shorter than in the Low-25(OH)D group, while there were no differences compared with the Normal-25(OH)D group. In addition, the cure rates were 86.7% in the Low-25(OH)D group and 96.7% in the two other groups. Taken together, the findings raise the possibility that calcitriol supplementation might elevate CD4^+^ T cell levels, shorten the time to sputum culture conversion, and accelerate lesion absorption in patients with TB who have 25(OH)D deficiency.

Previous epidemiologic studies demonstrated that 25(OH)D deficiency is associated with increased susceptibility to TB. Two systematic reviews and meta-analyses concluded that low serum 25(OH)D levels were associated with a higher risk of TB [12, 34]. Furthermore, other studies demonstrated that the serum 25-(OH) D levels of patients with TB were significantly lower than those of controls without TB [13–15]. Furthermore, Desai et al. found that 25(OH)D insufficiency occurred in 97% of 85 patients with TB [35]. The above research findings suggest that 25(OH)D deficiency is a risk factor for TB. However, published data are inconclusive regarding the effects of calcitriol supplementation on the response to anti-TB treatment.

A hypothesis of the present study was that calcitriol supplementation in patients with 25(OH)D deficiency would improve immune function by enhancing the levels of CD4^+^ T cells. Therefore, we measured the serum 25(OH)D levels and CD4^+^ T cell counts at baseline and at various time points during therapy. Importantly, we dynamically monitored the 25(OH)D levels in the Low-25(OH)D-Calcitriol group so that we could adjust the calcitriol dosage to maintain the serum 25(OH)D levels within the normal range. We found that the baseline CD4^+^ T cell count was significantly lower in patients with 25(OH)D deficiency than in patients who had normal levels of 25(OH)D. Furthermore, calcitriol supplementation resulted in a substantial increase in CD4^+^ T cell count that mirrored the change in serum 25(OH)D levels. Our results indicate that correcting 25(OH)D deficiency in patients with TB could lead to an improvement in CD4^+^ T cell count. Prior research in HIV-positive adults indicated that 25(OH)D deficiency could hasten the decline in CD4^+^ T cell counts [30] and that calcitriol supplementation during antiretroviral therapy could improve CD4^+^ T cell count [31, 36]. Furthermore, higher CD4^+^ T cell counts were associated with higher 25(OH)D levels in HIV-infected children [37]. The above-published data agree with our findings. Nevertheless, to the best of our knowledge, our study is the first to demonstrate that targeting calcitriol supplementation to patients with 25(OH)D deficiency can improve CD4^+^ T cell levels during the treatment of TB.

An important finding of the present study was that calcitriol supplementation in patients with 25(OH)D deficiency shortened the time to sputum culture conversion. Previous investigations yielded equivocal results regarding the effects of calcitriol supplements on sputum culture conversion. For example, some studies reported that calcitriol supplementation could enhance the rate of sputum culture conversion in patients with TB [38–40], shorten the time to sputum culture conversion in patients with multidrug-resistant pulmonary TB [17], and quicken the resolution of inflammatory responses during treatment for TB [40]. A meta-analysis by Wu et al. [16] showed that calcitriol supplementation increased the proportion of sputum smear and culture conversions, but not the time to conversion; they also showed that the supplementation increased the levels of lymphocytes. The above findings agree well with our data and suggest an accelerated resolution of the inflammatory responses during anti-tuberculosis therapy. Still, other studies have indicated that SNPs in 25(OH)D pathway genes influence the effect of 25(OH)D on time to sputum culture conversion, such that shortening of the time to sputum culture conversion was only observed in subsets of patients with particular SNPs (rs4334089, rs11568820, and rs731236 polymorphisms of the vitamin D receptor and rs4646536 polymorphism of 1α-hydroxylase) and not in the overall cohorts [18, 19, 41]. Furthermore, Daley et al. concluded that the administration of calcitriol did not shorten the time to sputum culture conversion [42], while Wejse et al. [43] and Tukvadze et al. [44] observed no effects of calcitriol on sputum smear conversion rates. It should be noted that the studies reporting negative effects of calcitriol administration on sputum smear conversion did not focus on patients with 25(OH)D deficiency, whereas our study examined the effects of calcitriol supplementation exclusively in patients with low 25(OH)D levels at baseline. Furthermore, unlike most previous studies, we measured 25(OH)D levels during treatment to confirm that calcitriol supplementation did correct the deficiency and avoid the possibility of giving too much calcitriol (which potentially could have detrimental effects on Th1 and Th2 cell function). In addition, by confirming differences in 25(OH)D levels between the Low-25(OH)D and Low-25(OH)D-Calcitriol groups, we were able to control for confounding factors that potentially could have affected 25(OH)D levels such as sunlight exposure and changes in diet. It is not unreasonable to assume that any beneficial effects of calcitriol would only be seen in patients with 25(OH)D insufficiency. Consistent with this proposal, Salahuddin et al. found that high-dose calcitriol accelerated clinical and radiographic improvement and enhanced the activation of the host immune system in those with low baseline levels of 25(OH)D [45]. Of course, other factors might also contribute to the different results among studies, including ethnicity, dose and formulation of the calcitriol supplement, and timing of the outcome evaluation. Regarding the latter factor, Nursyam et al. found that calcitriol supplementation increased the rate of sputum culture conversion at week 6 but not at later times, which suggests that the time to sputum culture conversion might be a more sensitive measure of the rate of clinical improvement during anti-TB therapy. Indeed, an important goal of TB treatment is to achieve sputum culture conversion as quickly as possible since this will help control the spread of the infection. It should also be noted that polymorphisms in the vitamin D receptor can affect its activity and hence the response to calcitriol supplementation [46]. Thus, vitamin D receptor SNPs might also contribute to inter-individual and inter-ethnicity variation in response to calcitriol supplementation.

Radiographic changes can also be used to assess the response to TB treatment. Although X-ray examinations can be helpful in the diagnosis and follow-up of TB, chest X-ray can miss some diagnoses and is unable to fully evaluate the location, morphology, distribution, density, size, edge, surrounding changes, and size of the lesions. Thus, low-dose spiral CT has become widely adopted as a safe and accurate method of imaging TB lesions. The present study demonstrated that calcitriol supplementation in patients with 25(OH)D deficiency shortened the time to 50% lesion absorption, which was evaluated by spiral CT. Consistent with our findings, Salahuddin et al. observed that lesion absorption at 12 weeks was greater for patients given intramuscular calcitriol than for those administered saline [45].

Although we found that calcitriol supplementation shortened both the time to sputum culture conversion and the time to 50% lesion absorption, there were no significant differences in cure rate among the three groups. Wejse et al. also observed no significant effect of calcitriol on the cure rate [43]. Based on the above results, we speculate that calcitriol supplementation in patients with 25(OH)D deficiency can accelerate recovery in those who respond to anti-TB treatment without increasing the number of patients who respond to therapy. Nevertheless, it should be noted that although there were no significant differences among the groups in our study, the cure rate was numerically lower in the Low-25(OH)D group than in the two other groups. Therefore, it cannot be excluded that our analysis had insufficient power to detect real differences among groups.

This study has several limitations. First, this was a single-center study, so the generalizability of the results is unknown. Second, the sample size was quite small, so the analysis may have been underpowered to detect some real differences among the groups. Third, the patients in the Normal-25(OH)D group were significantly younger than in the two other groups, and this might have confounded the results. Fourth, we did not investigate the possible influence of gene SNPs on the effects of calcitriol supplementation. Fifth, the present study only enrolled treatment-naïve and sputum smear-positive patients. Whether our findings are applicable to sputum smear-negative and retreated cases remains unknown. Sixth, it was beyond the scope of this study to elucidate the mechanisms by which calcitriol might influence CD4^+^ T cell levels. Finally, the definitions of normal 25(OH)D levels are conflicting [47, 48]. Studies have pointed out that healthy people need to keep 25(OH)D levels > 75 nmol/L to basically meet their health needs [49–51]. In this study, 25(OH)D < 75 nmol/L was considered low, and ≥ 75 nmol/L was considered normal, and deficiency was not distinguished.

## Conclusions

In conclusion, our findings suggest that calcitriol supplementation can elevate CD4^+^ T cell levels, shorten the time to sputum culture conversion, and accelerate lesion absorption in patients with TB who have 25(OH)D deficiency. Multicenter studies with large sample sizes are merited to confirm and extend our findings and establish the mechanism underlying the apparent link between 25(OH)D and CD4^+^ T cell count. Still, the results must be considered in the context in which the calcitriol group received only calcitriol treatment, while the low-vitamin D group did not receive vitamin D-related treatment. We consider that the supplementation of calcitriol increased the serum 25-hydroxyvitamin D3 content, but the mechanism is unclear. This mechanism will be further explored in the near future.

## Data Availability

All data generated or analyzed during this study are included in this published article.

## Abbreviations

TB: Tuberculosis
CT: Computed tomography
SNPs: Single nucleotide polymorphisms
WBC: White blood cell count
PLT: Platelet count
ALT: Serum levels of alanine transaminase
AST: Aspartate transaminase
CREA: Creatinine
SD: Standard deviation
ANOVA: Analysis of variance
